# Exploring the physical activity and screen-viewing-related knowledge, training, and self-efficacy of early childhood education candidates

**DOI:** 10.1186/s12887-018-1358-6

**Published:** 2019-01-05

**Authors:** Brianne A. Bruijns, Kristi B. Adamo, Shauna M. Burke, Valerie Carson, Jennifer D. Irwin, Patti-Jean Naylor, Brian W. Timmons, Leigh M. Vanderloo, Patricia Tucker

**Affiliations:** 1 Health and Rehabilitation Sciences Program, Faculty of Health Sciences, University of Western Ontario, London, Ontario Canada; 2 School of Human Kinetics, Faculty of Health Sciences, University of Ottawa, Ottawa, Ontario Canada; 3 School of Health Studies, Faculty of Health Sciences, University of Western Ontario, London, Ontario Canada; 4 Faculty of Kinesiology, Sport, and Recreation, University of Alberta, Edmonton, Alberta Canada; 5 School of Exercise Science, Physical and Health Education, University of Victoria, Victoria, British Columbia Canada; 6 Child Health and Exercise Medicine Program, McMaster University, Hamilton, Ontario Canada; 7 Child Health and Evaluative Science, Hospital for Sick Children, Toronto, Ontario Canada; 8 School of Occupational Therapy, Faculty of Health Sciences, University of Western Ontario, 1201 Western Road, Elborn College, Room 2547, London, ON N6G 1H1 Canada

**Keywords:** Physical activity, Early childhood education, Screen-viewing, Training, Young children

## Abstract

**Background:**

Early childhood educators greatly influence young children’s physical activity and screen-viewing behaviours in childcare. However, educators have requested additional training in physical activity programming, and one logical place to provide this education is during their pre-service schooling. This study explored the physical activity and screen-viewing-related knowledge, training, and self-efficacy of early childhood education (ECE) candidates across Canada, to determine their confidence and ability to facilitate physical activity opportunities and limit screen-viewing among young children in childcare.

**Methods:**

Key program personnel at 61 (of 110) Canadian colleges/universities offering an ECE program agreed to participate in this cross-sectional study. An online survey (112 items; 9 domains), developed by experts using the Tailored Design Method, was administered via Qualtrics© to a sample of 1292 ECE candidates. Descriptive statistics, Mann-Whitney *U*-tests, and chi-square tests were used to report participant demographics and physical activity and screen-viewing-related knowledge (i.e., of physical activity and screen-viewing concepts), training (i.e., physical activity and screen-viewing courses/content received), and self-efficacy (i.e., to facilitate physical activity and limit screen-viewing in childcare) of candidates.

**Results:**

ECE candidates exhibited the least amount of knowledge regarding the impact of screen-viewing on physiological outcomes (i.e., blood pressure) in young children. Further, only 32.2 and 26.7% of candidates reported completing physical activity or screen-viewing courses during their post-secondary training, respectively. Candidates who completed one or more physical activity or screen-viewing courses exhibited significantly greater (*p* <.05) self-efficacy than those without such training related to ensuring children were engaging in adequate moderate-to-vigorous physical activity (MVPA). Confidence to limit screen time did not differ. Candidates reporting meeting national physical activity recommendations (i.e., 150+ minutes of MVPA/week) exhibited significantly greater (*p* <.05) physical activity-related self-efficacy than those not meeting these guidelines.

**Conclusions:**

Findings from this work highlight both the need for and the potential of supplementary physical activity and screen-viewing content in post-secondary ECE programs to benefit candidates’ knowledge and self-efficacy in these areas. Introducing this content at the post-secondary level will ensure that all early childhood educators are appropriately trained regarding physical activity and screen-viewing before entering a childcare-based profession, where they can positively influence young children’s health behaviours.

## Background

Young children in centre-based childcare are exhibiting low levels of physical activity, in particular of moderate-to vigorous-intensity, [1] and engaging in unhealthy levels of sedentary behaviours [2], specifically screen-viewing [3]. These levels of engagement are worrisome as physical activity is consistently associated with more favourable physical, cognitive, and psychosocial health outcomes [4–6]. Further, sedentary behaviours are noted as an independent risk factor for a number of health complications, including decreased psychosocial health and cognitive abilities [7, 8]. While a number of sedentary behaviours, like reading, drawing, and circle time, serve an important developmental purpose (e.g., early language and literacy development [9]), there is emerging evidence that prolonged sedentary time is unhealthy, and that excessive screen-based sedentary time is problematic [10]. These health trends are alarming, particularly given that health behaviours established in early childhood are likely to carry across the lifespan [11]. As such, wide-scale health promotion efforts are warranted to encourage the early development and uptake of positive physical activity behaviours and limited screen-based sedentary time. Given that a large number of Canada’s young children (approximately 80%) are enrolled in some form of childcare [12], spending a substantial portion of their time in this arrangement [13], the childcare environment represents a feasible platform to intervene.

The childcare environment has been recognized as influencing the health behaviours of preschoolers [14], and early childhood educators serve as important gatekeepers within this environment [15] because they are responsible for daily programming [16]. Considering that educators’ incorporation of active opportunities into their childcare programming is largely dependent upon both their physical activity training [17] and personal preferences [18], it is essential that they are provided with related education. This type of training has been noted to foster educators’ confidence in and their likelihood of leading physical activity opportunities for young children in their care [19]. Young children in childcare have demonstrated higher levels of moderate-to vigorous-intensity physical activity (MVPA) when their educators were trained in physical activity [17]. With sedentary behaviour and screen-viewing research only recently emerging in the childcare literature, evidence of the effectiveness of interventions targeting these behaviours is not yet conclusive [3]. While physical activity training through professional development has been explored [17, 20, 21], researchers and early childhood educators themselves have proposed this training would be more influential and better received in the post-secondary setting [22] where it can effectively target the entire educator population and foster this related knowledge and self-efficacy prior to their entry into the workforce.

A pilot study has assessed the physical activity knowledge, training, and self-efficacy of early childhood education (ECE) candidates in Ontario colleges [23], showing that 72.1% of ECE candidates reported not receiving physical activity-related education. To date, no national study has been conducted; this is important because training, accreditation, and licensing requirements differ by jurisdiction [24, 25]. As such, an analysis of the physical activity and screen-viewing training ECE candidates received during their college/university education nationwide was necessary. Building upon work by Martyniuk and Tucker [23], the purpose of the present study was to examine the knowledge (i.e., of important physical activity and screen-viewing documents and concepts), training (i.e., the physical activity and screen-viewing courses offered and concepts covered), and self-efficacy (i.e., confidence to facilitate active opportunities for preschoolers and limit screen-viewing) of ECE candidates across Canada. Provincial/territorial differences in training were also examined, where possible.

It was hypothesized that physical activity and screen-viewing-specific training would be perceived as lacking from the majority of ECE curricula, and that self-reported physical activity and screen-viewing-related self-efficacy levels would be low among ECE candidates. More specifically, it was hypothesized that, consistent with previous literature [18, 19] and the findings from Martyniuk and Tucker’s pilot study [23], ECE candidates’ self-efficacy would be higher if they had completed one or more physical activity or screen-viewing-related courses, or if they engaged in the recommended levels of physical activity themselves.

## Methods

### Study design and procedures

Cross-sectional in design, this study followed a similar protocol to Martyniuk and Tucker’s pilot study [23]. Recruitment and data collection took place between January and May 2018. Ethical approval was provided by the Non-Medical Research Ethics Board at The University of Western Ontario (REB# 110246) and respective college/university research ethics boards, as requested.

### College/university recruitment

All Canadian colleges/universities offering an ECE program were identified (*n* = 110) [26]. Request for participation was initially made via email to program personnel (e.g., program coordinator, chair of the program, etc.), with a reminder sent if no response was received within 2 weeks, followed by a phone call if colleges/universities did not respond by email.

### Participants

#### Recruitment and inclusion criteria

Students enrolled in an ECE program at a participating Canadian college/university were invited to participate, regardless of enrolment status (e.g., full-time/part-time), year in the program, or program type (e.g., certificate, diploma, or degree). Due to the exploratory nature of the study, a sample size calculation was not completed. Invitation to complete the online survey was disseminated through email or a program website by college/university program personnel. The voluntary completion of the survey indicated consent to participate. To maximize participation, a reminder email was circulated to candidates 3 weeks after the initial invitation email. Class sizes from each college/university were documented to calculate provincial/territorial and national response rates.

#### Instruments and tools

Available in both English and French, a modified version of the survey employed in the Ontario pilot study [23] was completed by study participants. This 112-item tool measured nine domains: 1. physical activity and screen-viewing-related courses completed/forthcoming and concepts covered (*n* = 4 items); 2. knowledge of the relationship between physical activity/screen-viewing and health (*n* = 15 items); 3. familiarity with physical activity and screen-viewing-related documents and guidelines (*n* = 14 items); 4. self-efficacy to lead physical activity opportunities and minimize screen-viewing in childcare (*n* = 17 items); 5. awareness of the role of early childhood educators in modeling behaviours (*n* = 2 items); 6. views regarding helpful resources and supplementary training (*n* = 12 items); 7. personal values regarding physical activity and screen-viewing (*n* = 34 items); 8. personal physical activity and screen-viewing behaviours (*n* = 5 items); and, 9. demographic characteristics (*n* = 9 items). For the 17 self-efficacy items, ECE candidates rated their confidence to facilitate physical activity opportunities for and limit screen-viewing among children in childcare on an 11-point self-efficacy scale (‘0 = cannot do at all’ to ‘10 = highly certain can do’). Candidates rated whether they knew of physical activity and screen-viewing concepts (15 items) on a 6-point scale, and answers ranged from ‘1 = strongly disagree’ to ‘6 = strongly agree’.

The survey differed from Martyniuk and Tucker’s [23] survey by including screen-viewing and sedentary behaviour concepts, adding additional knowledge and self-efficacy items, and providing updated national physical activity and screen-viewing document versions and guidelines. A number of physical activity and screen-viewing-related documents were used in the development of this modified survey [27–32], as well as provincial/territorial childcare policy documents and additional academic research articles. The Tailored Design Method [33] was used in the tool’s creation to enhance response rates; questions were designed to be relatable and interesting to survey respondents and participants were informed of how the results would benefit their future profession. ECE professionals reviewed this tool to establish logical validity.

#### Data analysis

All statistical analyses were completed in SPSS (version 25). Descriptive statistics were used to report demographic characteristics and the physical activity and screen-viewing knowledge, training, and self-efficacy of ECE candidates.

Mean scores and standard deviations were calculated for all knowledge (*n* = 15) and self-efficacy (*n* = 17) items. As the data were non-normally distributed (Shapiro-Wilk = 0.62), non-parametric tests were conducted. Mann-Whitney *U* tests were performed to compare candidates’ self-efficacy to facilitate physical activity opportunities and minimize screen-viewing in childcare, dependent on the following grouping variables: 1. if they reported completing any physical activity/screen-viewing-related courses (i.e., 1 or more); and, 2. their own physical activity habits (i.e., whether they reported engaging in sufficient physical activity as per the *Canadian Physical Activity Guidelines for Adults* [150 min of MVPA per week] [34]). Also using the grouping above, two chi-square tests were performed to compare candidates’ physical activity and screen-viewing-related knowledge. To account for familywise error within multiple comparisons, the Holm-Bonferroni method was applied to adjust the *p*-values [35].

## Results

School representatives at 61 (of 110) colleges/universities agreed to participate, and a total of 1292 ECE candidates (of a potential 8089 invited candidates) completed the survey (response rate of 16%). The mean age of participating candidates was 25.67 ± 8.65 years, about half were Caucasian (55.1%), and the majority were female (96.1%), and enrolled full-time (89.1%) in a diploma program (71.5%). Most ECE candidates (85.2%) were either in the first or second year of their respective ECE programs, and 89.0% had previous work, volunteer, or placement experience in a childcare setting. Refer to Table 1 for institutional and participant provincial/territorial variation, and Table 2 for complete participant demographics.

**Table 1:** Provincial and Territorial Institutional and Early Childhood Education Candidate Participation

Province/ Territory	Number of Institutions Contacted	Number of Participating Institutions	Institutional Participation (%)	Enrolment Number at Participating Institutions	Number of Participants	Response Rate (%)
British Columbia	20	12	60	1085	184	17
Alberta	11	6	55	637	76	12
Saskatchewan	5	2	40	78	66	85
Manitoba	4	3	75	99	51	52
Ontario	27	20	74	5073	554	11^a^
Québec	31	9	29	683	90	13
Nova Scotia	4	4	100	223	26	12
New Brunswick	1	1	100	161	44	27
Prince Edward Island	2	1	50	6	2	33
Newfoundland & Labrador	2	1	50	35	28	80
Yukon	1	1	100	4	4	100
Northwest Territories	1	1	100	5	5	100
Nunavut	1	0	0	–	–	–
TOTAL	110	61	55	8089	1292	16^a^

*Note.* Percentages were rounded to the nearest percent. ^a^Slightly higher response rate due to college non-reporting

**Table 2:** Early Childhood Education Candidates’ Demographic Information and Personal Activity Behaviours *(n* = 1292)

Participant Characteristic	*N*	%	Participant Characteristic	*N*	%
Sex			Type of Early Childhood Education Program		
Male	12	1.8	Certificate	190	16.5
Female	645	96.1	Diploma	768	71.5
Ethnicity			Degree	115	9.9
Caucasian	370	55.1	Other	13	2.0
African Canadian	12	1.8	Year of Study		
Aboriginal/First Nations	53	7.9	1	582	49.3
Hispanic	11	1.6	2	424	35.9
Asian	105	15.6	3	71	6.0
Arab	16	2.4	4	42	3.6
Other	64	9.5	Other	61	5.2
Enrolment Status			Experience Working in a Childcare Setting		
Full-time	1048	89.1	Yes	1055	89.0
Part-time	128	10.9	No	131	11.0
Minutes of weekly MVPA			Minutes of daily recreational screen-viewing		
<30	147	21.8	<60	104	15.4
30–59	206	30.6	60–100	197	29.2
60–89	99	14.7	101–149	117	17.3
90–119	80	11.9	150–199	94	13.9
120–149	65	9.7	200–239	67	9.9
150+	76	11.3	240+	96	14.2

*Note.* Column total per section may not always match the total number of participants due to skipped questions; *MVPA* moderate-to-vigorous physical activity

Overall, participants reported not meeting physical activity guidelines; only 11.3% of candidates self-reported engaging in a minimum of 150 min of MVPA per week, while 69.1% engaged in 60 min or less of MVPA per week (Table 2). Regarding screen-viewing, 61.9% of candidates self-reported engaging in less than 150 min (2.5 h) per day of recreational screen time, while 14.2% reported engaging in 4 h or more per day (Table 2).

### Early childhood education candidates’ physical activity and screen-viewing knowledge

When ECE candidates were asked about their familiarity with a number of physical activity and screen-viewing-related documents, the large majority (73.4%) of candidates had knowledge of their respective provincial/territorial childcare legislation (i.e., the document governing care requirements within their province/territory); however, they were largely unaware of other physical activity or sedentary behaviour-specific documents of relevance for young children (see Table 3). Of note, only 15.1% of candidates were familiar with the *Canadian 24-Hour Movement Guidelines for the Early Years* [27], whereas 36.9 and 17.0% of candidates had knowledge of its preceding documents, the *Canadian Physical Activity Guidelines for the Early Years* and the *Canadian Sedentary Behaviour Guidelines for the Early Years*, respectively.

**Table 3:** Early Childhood Education Candidates’ Familiarity with Physical Activity and Sedentary Behaviour-Related Documents

Document	Yes (%)	No (%)
Provincial/territorial childcare legislation	73.4	26.6
ParticipACTION Report Card on Physical Activity for Children and Youth	19.6	80.4
Position Statement on Active Outdoor Play	23.7	76.3
Canadian Physical Activity Guidelines for the Early Years	36.9	63.1
Canadian Sedentary Behaviour Guidelines for the Early Years	17.0	83.0
Canadian 24-Hour Movement Guidelines for the Early Years^a^	15.1	84.9
I have never heard of any of these documents	20.3	79.7

*Note.* ^a^ The *Canadian 24-Hour Movement Guidelines for the Early Years* were only released 3 months prior to the dissemination of this survey

ECE candidates in the present study, on average, reported high physical activity knowledge; however, they scored lower when rating their knowledge of screen-viewing concepts (Table 4). Of the 15 knowledge items (Table 4), the highest average score reported by candidates was related to the key features of gross motor development (*M* = 5.30, *SD* = .80), while the lowest knowledge score was for the link between screen-viewing and high blood pressure (*M* = 4.55, *SD* = 1.26). Chi-square tests revealed no significant (*p* >.05) associations between any of the knowledge items and ECE candidates’ physical activity course exposure (Table 4), nor whether they were meeting the physical activity guidelines (Table 5).

**Table 4:** Candidates’ Physical Activity and Screen-Viewing-Related Knowledge, Total Sample and by Frequency of Course Content

	Total Sample		No courses		1+ Courses		*X* ^*2*^	Gamma	Gamma *SE*	T	Adj. *p*†
	*M*	*SD*	*M*	*SD*	*M*	*SD*					
**Physical Activity-Related Knowledge Item**											
Key features of gross motor development	5.30	.80	5.24	.86	5.46	.66	8.89	.12	.08	1.60	.87
Age appropriate movement skills for children	5.22	.84	5.16	.87	5.37	.77	2.50	.08	.07	1.14	1.00
The link between physical activity and cardiovascular health	5.00	.98	4.94	1.02	5.12	.89	5.38	.08	.07	1.19	1.00
The link between physical activity and muscular health	5.04	.96	5.00	.98	5.14	.93	1.88	.05	.07	.69	.49
The link between physical activity and psychosocial health	5.05	.94	5.01	.96	5.16	.92	1.38	.07	.07	1.01	1.00
The link between physical activity and learning	5.26	.84	5.20	.87	5.42	.73	5.85	.13	.07	1.77	.77
The link between physical activity, brain development, and preparing children for learning at school	5.22	.87	5.18	.90	5.31	.80	2.19	.08	.07	1.07	1.00
The link between physical inactivity and type 2 diabetes	4.82	1.18	4.82	1.17	4.84	1.19	11.28	.07	.07	.95	1.00
My college/university training has helped me understand important information about young children’s physical activity needs	5.15	.96	5.03	1.02	5.38	.79	14.99	.12	.07	1.70	.80
I have the skills and abilities I need to support young children’s physical activity	5.17	.93	5.08	1.00	5.35	.77	.72	.04	.06	.70	.97
**Screen-Viewing-Related Knowledge Item**											
The link between screen-viewing and rates of childhood obesity	5.09	1.02	5.09	1.00	5.14	1.01	.09	−.02	.11	−.18	1.00
The link between screen-viewing and psychosocial health	4.98	1.05	4.98	1.02	5.03	1.08	1.88	.01	.10	.07	.95
The link between screen-viewing and cognition	4.94	1.03	4.93	1.01	5.05	1.06	.70	.06	.10	.64	1.00
The link between screen-viewing and high blood pressure	4.55	1.26	4.53	1.24	4.67	1.28	2.01	.07	.09	.85	1.00
The link between screen-viewing and irregular sleep patterns	4.96	1.12	4.96	1.08	5.02	1.11	.18	.01	.10	.13	1.00

*Note.* PA-related knowledge item comparisons are based on number of PA-related courses taken, SV-related knowledge item comparisons are based on number of SV-related courses taken. *M* mean, *SD* standard deviation, *SE* Standard Error, *‘Adj.’* Adjusted, †The Holm-Bonferroni Method was applied to adjust the *p*-values for each set of multiple comparisons

**Table 5:** Early Childhood Education Candidates’ Knowledge Based on Meeting the Physical Activity Guideline for Adults

Physical Activity-Related Knowledge Item	*X* ^*2*^	Gamma	Gamma *SE*	T	Adj. *p*^†^
Key features of gross motor development	1.61	.27	.25	1.28	1.00
Age-appropriate movement skills for children	2.67	.34	.21	1.86	.58
The link between physical activity and cardiovascular health	3.82	.31	.15	2.22	.26
The link between physical activity and muscular health	1.90	.14	.15	.95	1.00
The link between physical activity and psychosocial health	1.61	.10	.15	.69	1.00
The link between physical activity and learning	1.10	.15	.20	.80	1.00
The link between physical activity, brain development, and preparing children for learning at school	1.34	.17	.19	.95	1.00
The link between physical inactivity and type 2 diabetes	1.23	.12	.13	.91	1.00
My college/university training has helped me understand important information about children’s physical activity needs	1.72	−.10	.14	−.68	.50
I have the skills and abilities I need to support children’s physical activity	.84	.11	.17	.69	.99

*Note. SE* Standard Error; †The Holm-Bonferroni Method was applied to adjust the *p*-values for each set of multiple comparisons

### Physical activity and sedentary behaviour-related training

When asked about their physical activity and sedentary behaviour training during their college/university education, 550 ECE candidates (67.8%) indicated that they had not completed, nor anticipated completing, any physical activity-specific courses, while 586 candidates (73.3%) reported having no sedentary behaviour-specific courses (Table 6). According to provincial frequencies, Nova Scotia and Quebec had the highest percentage of candidates with some (at least one) physical activity courses, with rates of 68.4 and 66.7%, respectively (Table 7). Candidates from the Northwest Territories, Prince Edward Island, and Yukon exhibited the lowest rates, with no candidates having reported the completion of any physical activity-specific courses (Table 7). With regard to sedentary behaviour courses, provincial frequencies were generally low; Alberta had the highest percentage (45.5%) of candidates who reported some sedentary behaviour-specific courses, followed by Quebec (41.0%; Table 7). However, 86.9% of all ECE candidates reported having covered *some* physical activity and/or sedentary behaviour-related content in other mandatory course lessons; the large majority of candidates reported that physical activity-related concepts such as gross motor development (86.6%), active play (81.4%), outdoor risky play (69.0%), and physical activity (68.3%) were covered in ECE curricula (Table 6). Conversely, only 41.5 and 47.3% of candidates indicated having covered sedentary behaviour and screen viewing-related content in their academic training, respectively.

**Table 6:** Physical Activity and Sedentary Behaviour Training during Early Childhood Education Candidates’ College/University Education

**Physical Activity and Sedentary Behaviour Courses Completed/Forthcoming**				
**Topic**	**No courses**		**1+ courses**	
	***N***	**%**	***N***	**%**
Physical Activity (*n* = 811)	550	67.8	261	32.2
Sedentary Behaviour (*n* = 799)	586	73.3	213	26.7
**Concepts Covered in Mandatory and Elective Courses (** ***n*** ** = 810)**				
**Topic**	**Mandatory**		**Elective**	
	***N***	***%***	***N***	**%**
Physical education	367	45.3	78	9.7
Physical activity	553	68.3	64	7.9
Physical literacy	374	46.2	76	9.4
Gross motor development	703	86.6	33	4.1
Locomotor & non-locomotor movement	463	57.2	56	6.9
Outdoor risky play	559	69.0	63	7.8
Active play	661	81.4	34	4.2
Screen viewing	383	47.3	75	9.3
Sedentary behaviour	336	41.5	70	8.6
Appropriate sleep	437	54.0	59	7.3
No courses discussed these topics	106	13.1	73	9.0

**Table 7:** Physical Activity and Sedentary Behaviour-Related Courses Completed/Forthcoming by Province/Territory

	Physical Activity Courses Completed/Forthcoming				Sedentary Behaviour Courses Completed/Forthcoming			
Province	No Courses		1+ Courses		No Courses		1+ Courses	
	*N*	%	*N*	%	*N*	%	*N*	%
Alberta	34	61.8	21	38.2	30	54.5	25	45.5
British Columbia	88	68.2	41	31.8	89	69.5	39	30.5
Manitoba	23	53.5	20	46.5	29	69.0	13	31.0
New Brunswick	13	61.9	8	38.1	18	85.7	3	14.3
Newfoundland & Labrador	20	74.1	7	25.9	23	85.2	4	14.8
Northwest Territories	3	100	0	0	3	100	0	0
Nova Scotia	6	31.6	13	68.4	12	70.6	5	29.4
Ontario	281	75.5	91	24.5	287	78.6	78	21.4
Prince Edward Island	1	100	0	0	1	100	0	0
Quebec	13	33.3	26	66.7	23	59.0	16	41.0
Saskatchewan	40	66.7	20	33.3	38	63.3	22	36.7
Yukon	3	100	0	0	2	100	0	0

### Self-efficacy to instruct physical activity and limit screen-viewing in childcare

Across the 17 items, the highest average self-efficacy score was for ECE candidates’ ability to create a childcare environment that encourages active play (*M* = 8.43, *SD* = 1.77; Table 8), whereas the lowest average rating pertained to confidence in their ability to lead active play opportunities in challenging weather climates (e.g., rain, snow, heat; *M* = 7.24, *SD* = 2.44; Table 8). When comparing ECE candidates’ self-efficacy based on the number of physical activity courses they completed, candidates who reported taking one or more physical activity courses had significantly greater confidence (mean rank = 369.32) to ensure that children were engaging in adequate MVPA as per the *Canadian 24-Hour Movement Guidelines* (*p* = .035) than candidates who reported no physical activity-related training (mean rank = 326.53; Table 8).

**Table 8:** Candidates’ Physical Activity and Sedentary Behaviour-Related Self-Efficacy, Total Sample and by Frequency of Course Content

Item	Total Sample		No Courses		1+ Courses		Mean Rank		Mann-Whitney *U*	*z*	Adj. *p†*
	*M*	*SD*	*M*	*SD*	*M*	*SD*	No courses	1+ Courses			
**Self-Efficacy to Promote Physical Activity**											
Ensure children are engaging in adequate light physical activity	7.93	2.04	7.82	2.12	8.15	1.84	333.60	357.55	47,801.00	−1.53	.25
Ensure children are engaging in adequate moderate-to-vigorous physical activity	7.37	2.20	7.19	2.30	7.73	1.95	326.53	369.32	44,441.00	−2.70	.04*
Create an environment that encourages active play	8.43	1.77	8.33	1.83	8.65	1.60	326.80	359.14	45,461.00	−2.11	.14
Make good use of the environment and available equipment for play and physical activity	8.32	1.82	8.20	1.94	8.60	1.51	326.63	357.93	45,508.50	−2.03	.13
Create opportunities for outdoor risky play (e.g., tree climbing, less ‘hovering’ on the playground, balancing activities)	7.26	2.46	7.25	2.53	7.34	2.26	339.35	336.76	50,008.50	−.16	.87
**Self-Efficacy to Teach Physical Activity**											
Model appropriate physical activity/movement behaviours	8.27	1.86	8.17	1.94	8.52	1.64	329.44	361.60	46,027.00	−2.07	.23
Lead activities to improve children’s fitness development (e.g., cardiovascular endurance, muscular strength, flexibility, & coordination)	7.71	2.17	7.64	2.22	7.87	2.06	332.93	351.37	47,863.50	−1.17	.72
Teach about the relationship between physical activity and health	7.66	2.11	7.62	2.17	7.79	1.97	335.55	346.07	48,936.50	−.67	.51
Teach locomotor skills, traveling actions (jump, gallop, hop)	8.29	1.94	8.19	2.06	8.54	1.65	330.46	353.28	46,991.50	−1.48	.69
Teach play skills (bike riding, sliding, swinging, climbing)	8.02	2.10	7.93	2.23	8.26	1.82	333.06	352.64	47,803.00	−1.25	.84
Teach rhythm skills	7.73	2.16	7.65	2.23	7.92	1.99	330.54	348.75	47,025.50	−1.16	.49
Use a variety of methods that encourage physical activity	8.11	1.94	7.98	2.01	8.39	1.76	325.36	363.62	44,685.00	−2.46	.10
**Self-Efficacy to Overcome Barriers to Physical Activity**											
Facilitate active play for young children in a limited space	7.96	2.00	7.85	2.10	8.23	1.74	327.87	354.00	46,065.50	−1.68	.09
Lead outdoor active play opportunities even if I am tired	7.98	2.00	7.85	2.11	8.24	1.76	324.62	355.91	44,975.00	−2.02	.13
Lead active play opportunities in challenging weather climates (e.g., rain, snow, extreme heat)	7.24	2.44	7.11	2.55	7.58	2.15	328.14	357.99	45,940.00	−1.89	.12
**Self-Efficacy to Minimize Screen Viewing**											
Limit the amount of screen time children in my class engage in to less than 40 min per day (*2/3 of the daily recommendation)	8.21	2.41	8.20	2.39	8.36	2.38	331.33	346.60	42,530.00	−.962	.34
Minimize the use of screens as a reward for good behaviour	7.96	2.54	7.92	2.60	8.19	2.50	330.36	351.04	41,900.00	−1.29	.39

*Note. M* = mean; *SD* standard deviation, Mann-Whitney *U* comparisons were between those with (1+ Courses) and without (No Courses) physical activity/screen viewing courses. ‘Adj.’ = Adjusted; †The Holm-Bonferroni Method was applied to adjust the *p*-values for each set of multiple comparisons. *p < .05

ECE candidates’ own physical activity levels also had an influence on their self-efficacy; those candidates who identified as active in accordance with the Canadian adult physical activity guidelines had greater confidence (mean rank = 391.63 and 399.09, respectively) to both create an environment that encourages active play (*p* = .008) and to make good use of the environment and available equipment for physical activity and play (*p* = .005) than those not meeting the guidelines (mean rank = 322.55 and 321.57, respectively; Table 9). Candidates meeting the guidelines had greater confidence (mean rank = 385.62, 398.53, and 398.07, respectively) for all three items within the ‘self-efficacy to overcome barriers to physical activity’ item group, which focused on their ability to: 1. facilitate active play for young children in a limited space (*p* = .006); 2. lead outdoor active play opportunities even if I am tired (*p* = .000); and, 3. lead active play opportunities in challenging weather climates (e.g., rain, snow, heat; *p* = .002), than their less active counterparts (mean rank = 322.86, 319.32, and 322.87, respectively; Table 9).

**Table 9:** Early Childhood Education Candidates’ Self-Efficacy Based on Candidates Meeting the Physical Activity Guideline for Adults

Item	Mean Rank		Mann-Whitney *U*	*z*	Adjusted *p*†
	Meeting Guideline	Not Meeting Guideline			
**Self-Efficacy to Promote Physical Activity**					
Ensure children are engaging in adequate light physical activity (as per the Canadian guidelines)	365.83	330.48	20,115.00	−1.54	.12
Ensure children are engaging in adequate moderate-to-vigorous physical activity (as per the Canadian guidelines)	377.82	327.22	18,975.50	−2.19	.06
Create a childcare environment that encourages active play	391.63	322.55	17,546.50	−3.08	.01*
Make good use of the environment and available equipment for play and physical activity	399.09	321.57	16,979.50	−3.44	.01*
Create opportunities for outdoor risky play (e.g., tree climbing, less ‘hovering’ on the playground, balancing activities)	380.64	325.70	18,609.00	−2.38	.05
**Self-Efficacy to Teach Physical Activity**					
Model appropriate physical activity/movement behaviours	386.30	326.70	18,407.50	−2.62	.04*
Lead activities to improve children’s fitness development (e.g., cardiovascular endurance, muscular strength, flexibility, & coordination)	401.68	323.56	17,086.00	−3.40	.01*
Teach about the relationship between physical activity and health	380.97	326.23	18,660.00	−2.38	.05
Teach locomotor skills, traveling actions (jump, gallop, hop)	395.96	323.14	17,369.00	−3.24	.01*
Teach play skills (bike riding, sliding, swinging, climbing)	375.07	327.57	19,184.50	−2.08	.08
Teach rhythm skills	349.22	327.53	20,458.50	−.945	.35
Use a variety of methods that encourage physical activity	398.72	322.78	17,159.50	−3.34	.01*
**Self-Efficacy to Overcome Barriers to Physical Activity**					
Facilitate active play for young children in a limited space	385.62	322.86	17,728.50	−2.75	.01*
Lead outdoor active play opportunities even if I am tired	398.53	319.32	16,718.00	−3.50	.00*
Lead active play opportunities in challenging weather climates (e.g., rain, snow, extreme heat)	398.07	322.87	17,209.00	−3.26	.00*

*Note.* Not meeting guideline indicates < 150 min of moderate-to-vigorous physical activity per week (CSEP, 2012b). Meeting guideline indicates ≥150 min of moderate-to-vigorous physical activity per week (CSEP, 2012b). †The Holm-Bonferroni Method was applied to adjust the *p*-values for each set of multiple comparisons. *p < .05

## Discussion

The purpose of this study was to explore the physical activity and screen-viewing knowledge, training, and self-efficacy of ECE candidates across Canada to better understand their confidence in and ability to promote physical activity and limit screen time among young children in childcare. This was the first study to provide a cross-provincial/territorial picture of the physical activity and screen-viewing educational experience of ECE candidates in Canada, a first step to understanding if there is a subsequent need for intervention to better serve this population before them entering a childcare-based profession. Multiple findings from this work warrant discussion.

While ECE candidates in the present study, on average, reported high physical activity knowledge, they scored lower when rating their knowledge of screen-viewing concepts. This finding is likely due to screens being frequently used as a pedagogical approach (i.e., media-based learning is increasingly prevalent and regarded as an effective educational tool [36]), as well as the infancy of this field of study; screen time recommendations for young children were only introduced in the past 6 years [37]. As such, appropriate screen-viewing behaviours for young children may not yet be integrated into the ECE curriculum, representing an opportunity to enhance training in post-secondary programs. These findings may warrant consideration from colleges/universities and childcare centres alike, as it is important for both curricula and policies to be evidence-informed. Offering early childhood educators supplementary course content and training in their post-secondary education would ensure this evidence is effectively integrated into their professional learning, thus better serving their development of practical knowledge and self-efficacy that can be used in their profession.

With the provision and facilitation of active opportunities for preschoolers being largely dependent upon early childhood educators’ physical activity training [17] and personal preferences [18], it is critical that they be appropriately trained regarding young children’s activity behaviours. Unfortunately, results from the present study revealed that only 32.2 and 26.7% of candidates reported completing or anticipated completing physical activity and sedentary behaviour courses during their post-secondary education, respectively. These results mirror the findings from Martyniuk and Tucker’s pilot study [23], where 27.9% of candidates reportedly had taken physical activity-specific courses. Conversely, most (86.9%) ECE candidates in the current study indicated that they had received some physical activity and/or sedentary behaviour content in other courses; however, some concepts (e.g., gross motor development, active play) were covered more frequently than others (e.g., sedentary behaviour, physical education), confirming that variability still exists among Canadian colleges/universities regarding the amount and comprehensiveness of such training. Inconsistent findings were not unexpected as each province/territory is regulated differently, both in terms of post-secondary education and childcare legislation. Interestingly, Nova Scotia (one of the three provinces/territories that actually stipulates a physical activity time requirement in its childcare regulation [24]) had the highest percentage of candidates (68.4%) with some physical activity-specific training. Noting the influence of policy on young children’s physical activity, Finch and colleagues [38] implemented an intervention in Australia to support childcare centres’ adoption of physical activity promoting policies and practices. Of the 228 centres in the intervention group, a significant increase in centres adopting a written physical activity policy (28% increase; *p* = < 0.01), as well as having staff trained in physical activity (47% increase; *p* = < 0.01), was observed post-intervention [38]. While centre-based policies and interventions have great potential, Ott et al. [39] reported that only 44% of Canadian childcare centres had a written physical activity policy, and very few had a policy surrounding physical activity training for staff. If physical activity policies for childcare centres were introduced at the provincial/territorial level (as is the case in Nova Scotia), perhaps colleges/universities would be inclined to integrate physical activity training into their curriculum design in order to address this requirement. Fostering such knowledge and confidence, which positively influences behaviour [40, 41], would be expected to produce graduates better able to carry out these policies in childcare settings.

Another finding from the current study that warrants discussion is ECE candidates’ lack of familiarity with various physical activity and sedentary behaviour-related documents. Notably, only 15.1% of candidates had heard of the *Canadian 24-Hour Movement Guidelines for the Early Years (0–4 years)*; however, this document was released only 3 months before the initial dissemination of the survey. Nevertheless, only 36.9 and 17.0% of candidates were familiar with its preceding documents, the *Canadian Physical Activity Guidelines for the Early Years* and the *Canadian Sedentary Behaviour Guidelines for the Early Years*, respectively, indicating that the majority of candidates may not be familiar with appropriate movement behaviour guidelines for young children. This suggests the need for more targeted sharing of physical activity guidelines among childcare professionals, as these individuals are responsible for the programming offered in childcare centres and ideally, the programming would align with these movement requirements. In contrast, most (73.4%) ECE candidates were familiar with their respective provincial/territorial childcare legislation. As such, if childcare legislation integrated components of the *Canadian 24-Hour Movement Guidelines* (e.g., scaling movement time recommendations to fit a childcare day), it is more likely this information would be relayed to candidates during their training. Duffey and colleagues [42] conducted a study to examine how well U.S. state childcare regulations incorporated national physical activity recommendations from the Institute of Medicine and found that the average number of recommendations included was 4.1 (*SD* = 1.4) out of 15. Interestingly, 40% of states had regulations regarding the amount of screen time allowed, whereas just 7% of states stipulated appropriate time spent in physical activity [42]. These authors agreed that state childcare policies should be more consistent with national physical activity recommendations in order to promote appropriate physical activity and screen-viewing behaviours in early learning settings.

The integration of physical activity and screen-viewing content into the ECE curriculum may prove beneficial, as previous studies have linked physical activity training to early childhood educators’ self-efficacy to facilitate active opportunities for young children in childcare [15]. In the present study, candidates who reportedly completed physical activity and screen-viewing courses scored significantly higher than those reporting being without this training regarding their confidence to ensure children were engaging in adequate levels of MVPA as per the Canadian guidelines. It seems logical that candidates with increased physical activity training scored higher on this item, as knowing what activities are considered MVPA and how to incorporate these into daily programming requires physical activity-specific knowledge. Trost and colleagues [43] conducted a review to examine how childcare policies and the environment impacted preschoolers’ physical activity. The authors found that staff education, training, and behaviours were strong predictors of children’s MVPA. With staff training being such a strong influence on young children’s MVPA [43], it is important to effectively prepare early childhood educators with related education. In Ontario, the College of Early Childhood Educators’ *Code of Ethics and Standards of Practice* stipulates that educators must “promote regular, healthy physical activity in all children” [44]. As such, related education in ECE candidates’ post-secondary program should be present. A review by Peden and colleagues [45] regarding early childhood educators’ physical activity training via professional learning indicated that while no clear length, mode, or content of such training proved superior, an exploration into multi-modal forms of professional learning (e.g., a combination of online and face-to-face training) may be more effective. Goldfield and colleagues [22] suggest that the college/university setting would serve as a feasible platform for this initiative.

Regardless of whether they reported completing physical activity courses, ECE candidates exhibited some of the lowest self-efficacy scores for all ‘overcoming barriers to physical activity’ items; this may indicate that practical instruction is generally lacking across all ECE programs. This gap in training is an important concern, as van Zandvoort and colleagues [46] conducted focus groups with early childhood educators (*n* = 54) and found that inadequate equipment, insufficient space, safety concerns, daycare requirements, and weather were all recurrent barriers to facilitating physical activity opportunities for young children in their care. Overcoming barriers to physical activity in early learning environments may be one way to effectively support increased physical activity and limited sedentary time among young children in these settings. Yet, if early childhood educators lack the training and resources to do so, achieving this goal may be challenging, and potentially result in active play being displaced by lower intensity or sedentary experiences.

While providing early childhood educators with additional training and resources may help increase their confidence and the likelihood of incorporating more physical activity into their programming, it is important to recognize the influence of early childhood educators’ own physical activity levels on their physical activity-related self-efficacy. ECE candidates who reported to be sufficiently active as per the Canadian physical activity guidelines had significantly greater physical activity-related self-efficacy than those not meeting the guidelines for 10 of the 17 self-efficacy items rated. It is concerning, then, that only 11.3% of candidates reported achieving the recommended level of MVPA per week. Of noted importance, in van Zandvoort and colleagues’ [46] previously described study, the early childhood educators reported that the more active they were at childcare, the more active they perceived the children in their care to be. Similarly, recent studies by Bell et al. [47] and Hesketh et al. [48] reported increased physical activity of young children in childcare when early childhood educators were active alongside them. While it may not be necessary for early childhood educators to meet physical activity recommendations, it is important they are cognizant of the strong influence they can have on young children’s movement behaviours.

The implications of this study’s results also extend beyond the ECE profession. Just as ECE professionals should be made aware of movement behaviour guidelines for young children, it is also important for pediatricians to be cognizant of these recommendations. Bearing in mind the abundant health benefits of physical activity [4–6] and consequences of excessive screen-based sedentary time [7, 8], knowledge of appropriate levels of these behaviours for this young population may influence pediatricians to advise parents and guardians to promote a physically active lifestyle. Pediatricians play an important health promotion role and increasing their awareness of movement behaviour guidelines may lead to these guidelines being integrated into their clinical practice; consideration of this targeted sharing of guidelines by health care policy-makers is warranted.

### Study limitations

Despite the multitude of important findings from this study, limitations must be considered. First, the exploratory nature of this study means that no causal inferences can be drawn. Additionally, despite efforts to recruit as many ECE candidates as possible, the nationwide response rate was only 16%, possibly biasing the sample; while typical online survey response rates tend to be lower than paper surveys, rates as high as 33% have been reported in the literature [49, 50]. Important to note, however, is that college and university students may be less inclined to participate in online surveys due to a multitude of factors, including survey length and being overrun with the educational demands of their program [51]. Future exploration of this topic may benefit from condensing knowledge, training, and self-efficacy items and/or administering the survey in shorter segments. Additionally, the majority of participating colleges/universities disseminated the survey recruitment email at the end of the term (a busy time for students), which may have also affected response rates. Low provincial/territorial response rates prevented exploring statistical inferences and may also limit the within-province/−territory generalizability of findings. Despite lower than anticipated participation, the large overall sample size of 1292 provides a preliminary understanding of the physical activity and screen-viewing-related education provided to ECE candidates in Canadian post-secondary programs.

The self-report nature of the survey is also a limitation, as the data collected reflects ECE candidates’ retention of course concepts and knowledge rather than actual content covered in the curriculum. The survey length also resulted in incomplete data, so questions later in the survey produced a lower response rate. Furthermore, candidates may have been subject to social desirability bias, as some survey questions may have been leading or candidates may have felt pressured to select a more desirable answer. Finally, volunteer bias may have been present for colleges who opted to offer candidates class time to complete the survey, and even though participation was voluntary and anonymous, the presence of the professor may have created undue influence on candidates to participate.

## Conclusion

This study provides a preliminary indication of the physical activity and screen-viewing knowledge, training, and self-efficacy of ECE candidates nationwide. The majority of ECE candidates reported not receiving any physical activity or screen-viewing-related education; however, those who did report receiving such training exhibited greater self-efficacy to engage young children in MVPA. It remains unclear whether practical skills are being transmitted in ECE programs; in fact, with low barrier self-efficacy demonstrated by ECE candidates in the present study overall, this may not be the case. As such, more research is needed and clearly interventions are the next step. Future research should explore ECE course instructors’ reported curriculum to determine if it matches content reported by their students. Additionally, considering provincial comparisons were unable to be conducted in the present study, gathering this information would provide Ministries of Education with more comprehensive evidence to consider when developing curriculum requirements. Moreover, piloting supplementary physical activity and screen-viewing training in select Canadian ECE programs would provide useful information about whether this addition would be effective at increasing candidates’ physical activity and screen-viewing knowledge and self-efficacy. Findings from the present study may encourage provincial Ministries of Education, as well as college/university faculty and staff to consider making modifications to current ECE curricula requirements and/or course content to ensure its trainees are well-prepared to support healthy movement behaviours among young children in childcare.

## Abbreviations

ECE: Early childhood education
MVPA: Moderate-to-vigorous physical activity
